# Supplementary material for: Noncanonical assembly, neddylation and chimeric cullin–RING/RBR ubiquitylation by the 1.8 MDa CUL9 E3 ligase complex
Source: Nat Struct Mol Biol. 2024 Apr 11;31(7):1083–94. doi: 10.1038/s41594-024-01257-y (PMC11257990; doi:10.1038/s41594-024-01257-y)
Supplement: Supplementary file 1 — Supplementary Tables 1–5. [file 41594_2024_1257_MOESM1_ESM.pdf]

nature structural & molecular biology

Article

https://doi.org/10.1038/s41594-024-01257-y

Noncanonical assembly, neddylation and chimeric cullin–RING/RBR ubiquitylation by the 1.8 MDa CUL9 E3 ligase complex

In the format provided by the authors and unedited

CROSSLINKED	RESIDUES	LOCATION WITHIN STRUCTURE	SCORE
Position 1	Position 2		
**188**	188	ARM1 | ARM1	166.16
**507**	64**(UBE2L3)**	ARM2 | **UBE2L3**	161.79
87	2285	Loop between SBD and ARM1 | Ariadne	154.56
87	188	Loop between SBD and ARM1 | ARM1	153.08
63**(UB)**	48**(UB)**	**ubiquitin | ubiquitin**	149.4
63**(UB)**	33**(UB)**	**ubiquitin | ubiquitin**	126.67
532	507	ARM2 | ARM2	113.62
1165	1086	DOC | ARM3	111.64
85**(UBE2L3)**	48**(UB)**	**UBE2L3 | ubiquitin**	105.95
33**(UB)**	48**(UB)**	**ubiquitin | ubiquitin**	104.45
1289	48**(UB)**	DOC | **ubiquitin**	103.02
73**(UBE2L3)**	146**(UBE2L3)**	**UBE2L3 | UBE2L3**	94.05
1165	46	DOC | SBD	90.71
1165	1184	DOC | DOC	89.67
2230	2144	Rcat | RING1	87.84
1795	1718	C/R | C/R	73.93
48**(UB)**	2234	**ubiquitin** | Rcat	73.57
1503	25	CR3 | SBD	73.29
48**(UB)**	1879	**ubiquitin** | WHB	69.39
48**(UB)**	145**(UBE2L3)**	**ubiquitin | UBE2L3**	69.39
2144	1452	RING1 | CR3	66.54
1503	1452	CR3 | CR3	65.29
48**(UB)**	627	**ubiquitin** | Loop between ARM2 and ARM9	64.78
1184	2234	DOC | Rcat	63.29
1184	615	DOC | Loop between ARM2 and ARM9	63.15
1165	1452	DOC | CR3	62.57
33**(UB)**	2144	**ubiquitin** | RING1	61.01
986	9**(UBE2L3)**	ARM3 | **UBE2L3**	57.49
73**(UBE2L3)**	48**(UB)**	**UBE2L3 | ubiquitin**	57.28
48**(UB)**	1452	**ubiquitin** | CR3	55.85
986	48**(UB)**	ARM3 | **ubiquitin**	54.71
63**(UB)**	73**(UBE2L3)**	**ubiquitin | UBE2L3**	54.55
627	615	Loop between ARM2 and ARM9 | Loop between ARM2 and ARM9	54.54
1184	627	DOC | Loop between ARM2 and ARM9	49.36
2007	2144	UBAL | RING1	49.31
658	627	Loop between ARM2 and ARM9 | Loop between ARM2 and ARM9	48.5
48**(UB)**	1452	**ubiquitin** | CR3	47.81
105 **(RBX1)**	1452	C-term **(RBX1)** | CR3	47.81
63**(UB)**	2234	DOC | RING **(RBX1)**	47.81
131**(UBE2L3)**	48**(UB)**	**UBE2L3 | ubiquitin**	47.08
48**(UB)**	1184	**ubiquitin** | DOC	45.72

1184	146**(UBE2L3)**	DOC | **UBE2L3**	44.13
1184	89 **(RBX1)**	DOC | RING **(RBX1)**	42.91
1168	1452	DOC | CR3	41.52
1184	1452	DOC | CR3	41.19

Supplementary Table 1 - XL-MS analysis of the hexameric CUL9-RBX1 complex with UBE2L3~Ubiquitin

LYSINE RESIDUE	LOCATION WITHIN STRUCTURE	MS1 INTENSITY
87	Loop between SBD and ARM1	1.91E+10
627	Loop between ARM3 and ARM9	4.25E+9
1881	Neddylation site in WHB domain	4.12E+9
620	Loop between ARM3 and ARM9	6.59 E+8
2144	RING1 domain	2.8E+8
615	Loop between ARM3 and ARM9	2.36E+8
1668	bridging helix	1.12E+8
1184	DOC domain	9.56E+7
1090	ARM3	5.66E+7
2141	RING1 domain	3.42E+7
2007	UBAL domain	3.07E+7
1191	DOC domain	2.16E+7
1299	DOC domain	1.09E+7
1657	bridging helix	6.21E+6

Supplementary Table 2 – DiGly-MS analysis of CUL9-RBX1 ubiquitylation sites

LYSINE RESIDUE	LOCATION WITHIN STRUCTURE
320	Nuclear localization signal (NLS)
321	NLS
319	NLS
292	DNA binding domain
357	Loop between NLS and oligomerization domain
386	C-terminal domain
24	transactivation domain

Supplementary Table 3 - DiGly-MS analysis of TP53 ubiquitylation sites by CUL9-RBX1

CROSSLINKED	RESIDUES	LOCATION WITHIN STRUCTURE	SCORE
Position 1	Position 2		
188	188	ARM1 | ARM1	278.27
523	507	ARM2 | ARM2	261.65
188	188	ARM1 | ARM1	245.93
1299	1165	DOC | DOC	122.74
87	188	ARM1 | ARM1	116.52
1165	1086	DOC | ARM3	116
87	2285	Loop between SBD and ARM1 | Ariadne	114.5
2230	2144	Rcat | RING1	110.63
188	188	ARM1 | ARM1	108.98
1184	615	DOC | Loop between ARM2 and ARM9	102.22
1668	87	Bridging helix | Loop between SBD and ARM1	95.42
1165	46	DOC | SBD	94.89
1299	46	DOC | SBD	93.26
1165	1184	DOC | DOC	89.67
1881	1868	WHB | WHB	89.35
761	627	ARM9 | Loop between ARM2 and ARM9	87.99
1299	1165	DOC | DOC	84.5
1184	1086	DOC | ARM3	83.84
1090	1184	ARM3 | DOC	83.84
1184	64 (UB)	DOC **| ubiquitin**	83.84
1184	2144	DOC | RING1	83.8
2230	2144	IBR | RING1	81
1165	1086	DOC | ARM3	77.75
1299	1086	DOC | ARM3	77.75
1503	25 **(RBX1)**	CR3 | N-terminus **(RBX1)**	76.29
1090	627	ARM3 | Loop between ARM2 and ARM9	76.15
89 **(RBX1)**	1452	RING **(RBX1)** | CR3	73.29
1503	1452	CR3 | CR3	73.29
1881	1868	WHB | WHB	69.39
1881	2144	WHB | RING1	69.39
2007	2144	UBAL | RING1	68.64
1881	2144	WHB | RING1	68.03
2007	2144	UBAL | RING1	67.38
1184	89 **(RBX1)**	DOC | RING **(RBX1)**	66.66
1503	25 **(RBX1)**	CR3 | N-terminus **(RBX1)**	66.54
1881	1868	WHB | WHB	66.44
2144	1452	RING1 | CR3	65.29
613	620	Loop between ARM2 and ARM9 | Loop between ARM2 and ARM9	65.25
1184	89 **(RBX1)**	DOC | RING **(RBX1)**	64.8

2230	2144	IBR | RING1	63.37
613	627	Loop between ARM2 and ARM9 | Loop between ARM2 and ARM9	60.96
627	615	Loop between ARM2 and ARM9 | Loop between ARM2 and ARM9	59.32
1184	1452	DOC | CR3	58.74
1184	2230	DOC | Rcat	57.56
1184	1086	DOC | ARM3	57.56
1184	1086	DOC | ARM3	57.56
1090	1184	ARM3 | DOC	57.56
2144	1452	RING1 | DOC	56.75
1165	46	DOC | SBD	54.55
627	615	Loop between ARM2 and ARM9 | Loop between ARM2 and ARM9	54.54
627	2144	Loop between ARM2 and ARM9 | RING1	54.54
2285	2267	Ariande | Rcat	53.55
48 **(UB)**	1184	**ubiquitin** | DOC	53.55
1503	25	CR3 | SBD	52.96
627	615	Loop between ARM2 and ARM9 | Loop between ARM2 and ARM9	52.68
1184	1168	DOC | DOC	51.99
2007	2144	UBAL | RING1	51.9
1718	1881	C/R | WHB	51.69
1184	627	DOC | Loop between ARM2 and ARM9	49.36
1090	1184	ARM3 | DOC	49.36
2007	6**(NEDD8)**	UBAL | **NEDD8**	48.24
1718	1881	C/R | WHB	47.91
89	1452	Loop between SBD and ARM1 | CR3	47.81
986	627	ARM3 | Loop between ARM2 and ARM9	46.89
1168	1452	DOC | CR3	46.4
761	627	ARM9 | Loop between ARM2 and ARM9	45.97
52	1452	SBD | CR3	44.13
1168	1452	DOC | CR3	43.99
1184	627	DOC | Loop between ARM2 and ARM9	43.03
2285	1184	Ariadne | DOC	42.82
2007	2144	UBAL	42.77
1299	1168	ARM3	42.46

Supplementary Table 4 - XL-MS analysis of the hexameric CUL9-RBX1 complex

CROSSLINKED	RESIDUES	LOCATION WITHIN STRUCTURE	SCORE
**POSITION 1**	Position 2		
**523**	507	ARM2 | ARM2	242.2
87	2285	Loop between SBD and ARM1 | Ariadne	136.16
48**(UB)**	**9(UBE2L3)**	**ubiquitin** | **UBE2L3**	121.87
1299	48**(UB)**	DOC | **ubiquitin**	109.63
9**(UBE2L3)**	20 **(UBE2L3)**	**UBE2L3**| **UBE2L3**	105.82
613	620	Loop between ARM2 and ARM9 | Loop between ARM2 and ARM9	104.45
2230	2144	Rcat | RING1	103.7
135**(UBE2L3)**	146**(UBE2L3)**	**UBE2L3** | **UBE2L3**	100.01
1165	46	DOC | SBD	94.89
1165	1086	DOC | ARM3	93.43
188	261	ARM1 | ARM2	93.18
1449	105(**RBX1)**	CR3 | C-term (**RBX1)**	88.59
87	188	Loop between SBD and ARM1 | ARM1	85.86
64 **(UBE2L3)**	86**(UBE2L3)**	**UBE2L3** | **UBE2L3**	84.17
1184	2144	DOC | RING1	73.21
87	986	Loop between SBD and ARM1 | ARM3	69.42
73**(UBE2L3)**	48	**ubiquitin** | Rcat	66.68
64**(UBE2L3)**	73**(UBE2L3)**	**UBE2L3**| **UBE2L3**	64.76
71**(UBE2L3)**	64**(UBE2L3)**	**UBE2L3**| **UBE2L3**	62.69
2007	2144	UBAL | RING1	61.32
131**(UBE2L3)**	138**(UBE2L3)**	**UBE2L3**| **UBE2L3**	60.79
1184	1086	DOC | ARM3	60.79
9 **(UBE2L3)**	16 **(UBE2L3)**	**UBE2L3** | **UBE2L3**	55.06
48**(UB)**	138 (**UBE2L3)**	**ubiquitin** | **UBE2L3**	53.16
2144	2234	RING1 | Rcat	52.36
627	615	Loop between ARM2 and ARM9 | Loop between ARM2 and ARM9	51.9
48**(UB)**	145 **(UBE2L3)**	**ubiquitin** | **UBE2L3**	51.04
136**(UBE2L3)**	145**(UBE2L3)**	**UBE2L3**| **UBE2L3**	50.16
188	986	ARM1 | ARM3	50.12
105**(RBX1)**	1452	C-term **(RBX1)** | CR3	49.44
2285	2267	Ariadne | Rcat	47.74
1090	1184	ARM3 | DOC	47.74
1165	1452	DOC | CR3	46.4
1299	1165	CR2 | DOC	46.22
2144	1452	RING1 | CR3	44.13
986	1184	ARM3 | DOC	43.12
145**(UBE2L3)**	146**(UBE2L3)**	**UBE2L3**| **UBE2L3**	41.43
145**(UBE2L3)**	150**(UBE2L3)**	**UBE2L3**| **UBE2L3**	40.69

Supplementary Table 5- XL-MS analysis of the monomeric CUL9-RBX1 complex with UBE2L3~Ubiquitin
