# Supplementary material for: Noncanonical assembly, neddylation and chimeric cullin–RING/RBR ubiquitylation by the 1.8 MDa CUL9 E3 ligase complex
Source: Nat Struct Mol Biol. 2024 Apr 11;31(7):1083–94. doi: 10.1038/s41594-024-01257-y (PMC11257990; doi:10.1038/s41594-024-01257-y)
Supplement: Supplementary file 2 — Reporting Summary [file 41594_2024_1257_MOESM2_ESM.pdf]

nature portfolio

Corresponding author(s): Brenda A. Schulman

Last updated by author(s): Jan 29, 2024

Reporting Summary

Nature Portfolio wishes to improve the reproducibility of the work that we publish. This form provides structure for consistency and transparency in reporting. For further information on Nature Portfolio policies, see our Editorial Policies and the Editorial Policy Checklist.

Statistics

For all statistical analyses, confirm that the following items are present in the figure legend, table legend, main text, or Methods section.

n/a	Confirmed	
☐	☒	The exact sample size (*n*) for each experimental group/condition, given as a discrete number and unit of measurement
☐	☒	A statement on whether measurements were taken from distinct samples or whether the same sample was measured repeatedly
☐	☒	The statistical test(s) used AND whether they are one- or two-sided<br>*Only common tests should be described solely by name; describe more complex techniques in the Methods section.*
☒	☐	A description of all covariates tested
☒	☐	A description of any assumptions or corrections, such as tests of normality and adjustment for multiple comparisons
☐	☒	A full description of the statistical parameters including central tendency (e.g. means) or other basic estimates (e.g. regression coefficient) AND variation (e.g. standard deviation) or associated estimates of uncertainty (e.g. confidence intervals)
☐	☒	For null hypothesis testing, the test statistic (e.g. F, t, r) with confidence intervals, effect sizes, degrees of freedom and P value noted<br>*Give P values as exact values whenever suitable.*
☒	☐	For Bayesian analysis, information on the choice of priors and Markov chain Monte Carlo settings
☒	☐	For hierarchical and complex designs, identification of the appropriate level for tests and full reporting of outcomes
☒	☐	Estimates of effect sizes (e.g. Cohen's d, Pearson's r), indicating how they were calculated

Our web collection on statistics for biologists contains articles on many of the points above.

Software and code

Policy information about availability of computer code

Data collection	Gel imaging: Amersham Imager 600, Amersham Typhoon; Cryo-EM: SerialEM v3.8.0-b5, FEI EPU v2.7.0
Data analysis	Assay Analysis: GraphPad Prism v9.2.0; Cryo-EM: RELION v3.1, RELION 4.0, Gautomatch v0.56, CTFFIND v4.1; Structure Analysis and Visualization: Chimera v1.13.1, ChimeraX v1.2.5; Model Building: COOT v0.8.9.1, Phenix.refine v1.17.1, DeepEMhancer version 2020.09.07 (https://github.com/rsanchezgarc/deepEMhancer); SEC-MALS: Wyatt Technology ASTRA v5.3; Massphotometry: Refeyn DiscoverMP v2.3.0; Mass spectrometry: Proteome Discoverer v2.5.0.400, cross-link analyzer v1.1.4, MaxQuant v2.2.0.0, MaxQuant v1.6.2.10, Python version v3.5.5 with packages numpy v1.21.5 and pandas v1.4.2.

For manuscripts utilizing custom algorithms or software that are central to the research but not yet described in published literature, software must be made available to editors and reviewers. We strongly encourage code deposition in a community repository (e.g. GitHub). See the Nature Portfolio guidelines for submitting code & software for further information.

Data

Policy information about availability of data

All manuscripts must include a data availability statement. This statement should provide the following information, where applicable:

- Accession codes, unique identifiers, or web links for publicly available datasets
- A description of any restrictions on data availability
- For clinical datasets or third party data, please ensure that the statement adheres to our policy

Cryo-EM maps will be available from the Electron Microscopy Data Bank, and the model coordinates will be available from Protein Data Bank upon publication: PDB ID 8Q7H (focused neddylated and unneddylated cullin dimer), PDB ID 8Q7E (hexameric assembly), PDB ID 8RHZ (unneddylated cullin dimer built in symmetry expanded map) and EMDB with codes EMD-18216 (focused neddylated and unneddylated cullin dimer), EMD-18214 (hexameric assembly), EMD-19179 (unneddylated cullin dimer symmetry expanded map), EMD-18218 (focused dimeric core), EMD-18217 (focused on E2-like density), EMD-18220 (CUL9ΔCPH-RBX1), EMD-18222 (CUL9ΔARM9-RBX1), EMD-18223 (CUL9ΔARIH-RBR-RBX1), EMD-18221 (CUL9ΔDOC-RBX1). The mass spectrometry data have been deposited to the ProteomeXchange Consortium (http://proteomecentral.proteomexchange.org) via the PRIDE repository with the dataset identifier PXD047326, PXD047229. Raw gels are provided as source data. Accession codes of published data which was used for comparison: PDB: 8Z7B, 7B5L, 1LDJ, 6V9I, 7ONI, 4P5O, 6R7N.

Research involving human participants, their data, or biological material

Policy information about studies with human participants or human data. See also policy information about sex, gender (identity/presentation), and sexual orientation and race, ethnicity and racism.

Reporting on sex and gender	N/A
Reporting on race, ethnicity, or other socially relevant groupings	N/A
Population characteristics	N/A
Recruitment	N/A
Ethics oversight	N/A

Note that full information on the approval of the study protocol must also be provided in the manuscript.

Field-specific reporting

Please select the one below that is the best fit for your research. If you are not sure, read the appropriate sections before making your selection.

☒ Life sciences ☐ Behavioural & social sciences ☐ Ecological, evolutionary & environmental sciences

For a reference copy of the document with all sections, see nature.com/documents/nr-reporting-summary-flat.pdf

Life sciences study design

All studies must disclose on these points even when the disclosure is negative.

Sample size	Sample size calculations were not performed. Selected sample sizes were designed to ensure clear and reliable interpretation of the results. Based on previous experience in terms of variability, at least two independent replicates were carried out for all functional assays.
Data exclusions	No data were excluded.
Replication	All experiments were performed at least twice, with numerous controls. All attempts at replication were successful.
Randomization	No grouped samples.
Blinding	No grouped samples.

Reporting for specific materials, systems and methods

We require information from authors about some types of materials, experimental systems and methods used in many studies. Here, indicate whether each material, system or method listed is relevant to your study. If you are not sure if a list item applies to your research, read the appropriate section before selecting a response.

Materials & experimental systems

n/a	Involved in the study	
☐	☒	Antibodies
☐	☒	Eukaryotic cell lines
☒	☐	Palaeontology and archaeology
☒	☐	Animals and other organisms
☒	☐	Clinical data
☒	☐	Dual use research of concern
☒	☐	Plants

Methods

n/a	Involved in the study	
☒	☐	ChIP-seq
☒	☐	Flow cytometry
☒	☐	MRI-based neuroimaging

Antibodies

Antibodies used

The antibodies against NEDD8 (#2745), UBE2M (#4913), β-Actin (#4967) were from Cell Signaling Technology. The antibodies against UBE2F (sc-398668) were from Santa Cruz Biotechnology. Anti-CUL9 antibody was a kind gift from Arno Alpi; this antibody was raised and validated by the MRC PPU Reagents and Services, School of Life Sciences, University of Dundee, Dundee, Scotland, DD1 5EH. The antibody against Vinculin (ab129002) was obtained from Abcam.

Validation

NEDD8 (https://www.cellsignal.com/products/primary-antibodies/nedd8-antibody/2745?_requestid=2891057)
UBE2M (https://www.cellsignal.com/products/primary-antibodies/ubc12-antibody/4913)
β-Actin (https://www.cellsignal.com/products/primary-antibodies/b-actin-antibody/4967)
UBE2F (https://www.scbt.com/de/p/ube2f-antibody-c-11)
CUL9 (https://mrcppureagents.dundee.ac.uk/ and this study)
Vinculin (https://www.abcam.com/products/primary-antibodies/vinculin-antibody-epr8185-ab129002.html)

Eukaryotic cell lines

Policy information about cell lines and Sex and Gender in Research

Cell line source(s)

HEK293S GnTI- (identifier: CRL-3022), U2OS (identifier: HTB-96) were obtained from ATCC. U2OS CUL9 knockout cell line was a kind gift by Yue Xiong. Sf9 cells were obtained from Thermo Fischer (identifier: 11496015).

Authentication

Cell lines were not authenticated.

Mycoplasma contamination

Cell lines were periodically tested for Mycoplasma contamination and were always negative.

Commonly misidentified lines
(See ICLAC register)

No commonly misidentified cell lines were used in this study.
