# Supplementary figures and images for: Noncanonical assembly, neddylation and chimeric cullin–RING/RBR ubiquitylation by the 1.8 MDa CUL9 E3 ligase complex
Source: Nat Struct Mol Biol. 2024 Apr 11;31(7):1083–94. doi: 10.1038/s41594-024-01257-y (PMC11257990; doi:10.1038/s41594-024-01257-y)

Figure 1

f

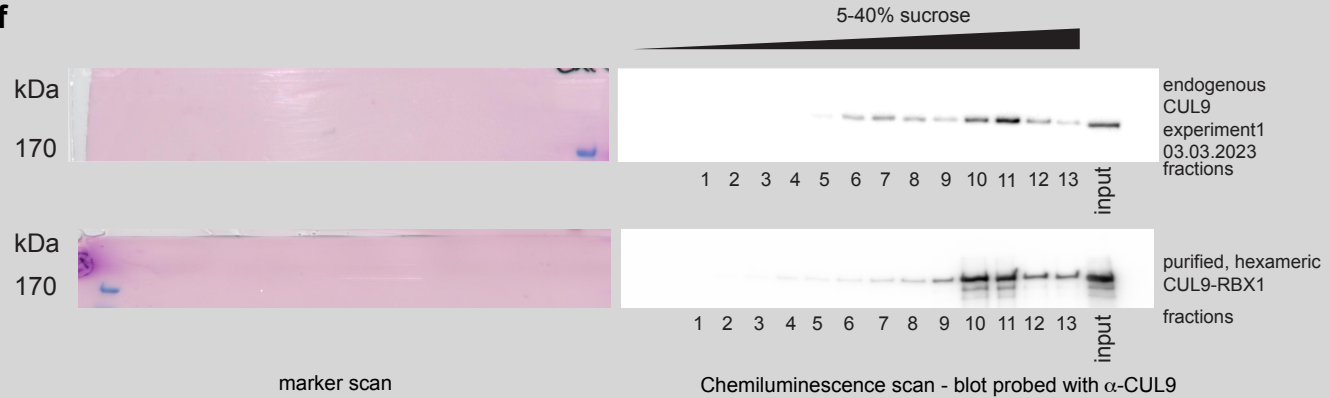

Supplement: Supplementary file 5 — Unprocessed western blots and/or gels. [file 41594_2024_1257_MOESM5_ESM.pdf]

**Figure 3**

**b**

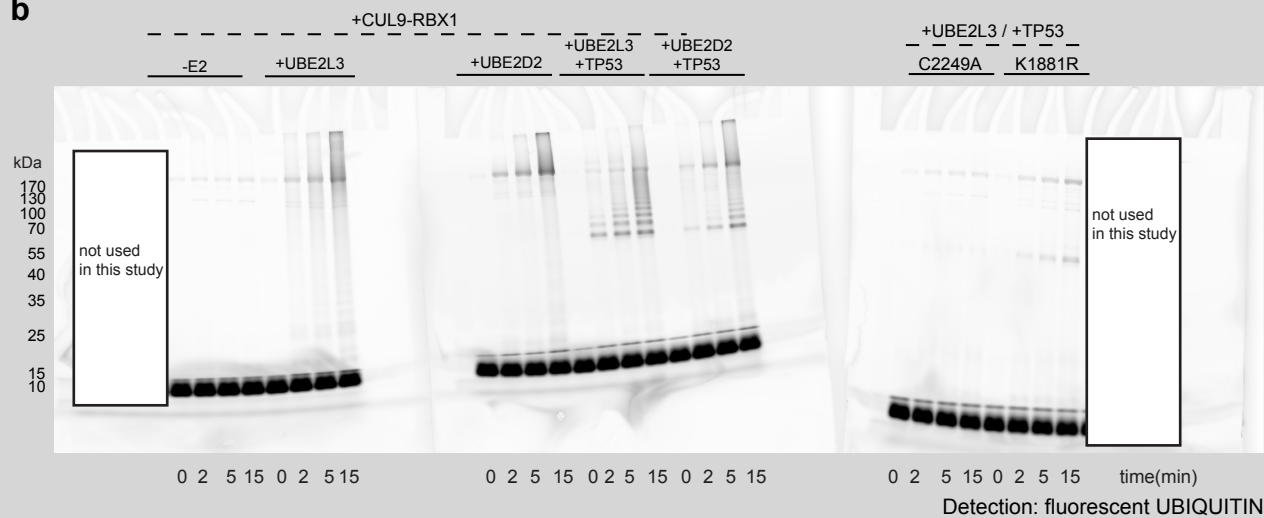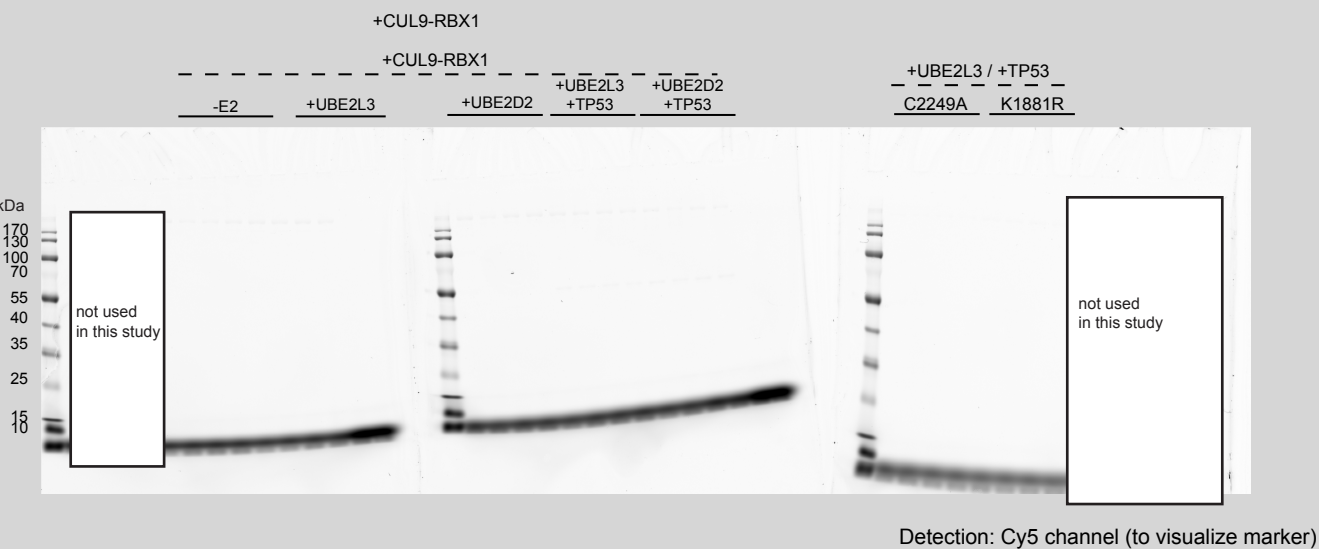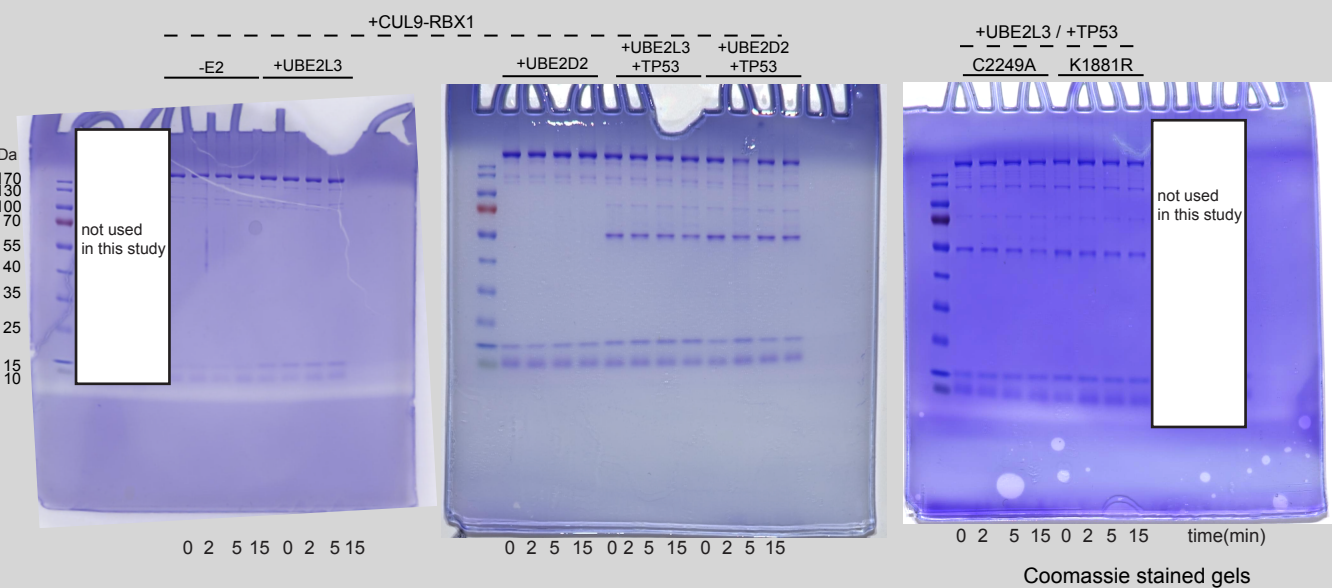

Figure 3  
h

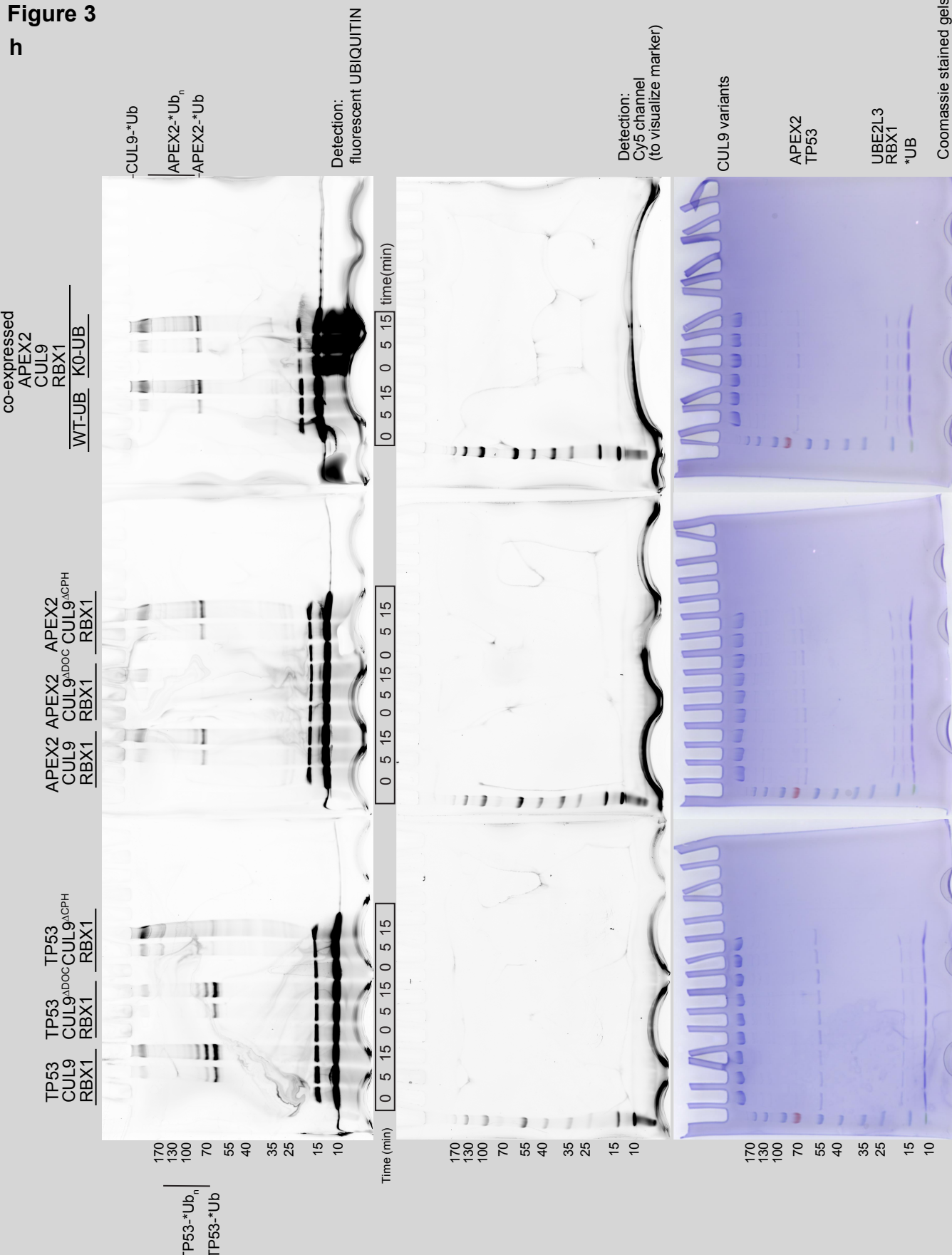

Supplement: Supplementary file 6 — Unprocessed western blots and/or gels. [file 41594_2024_1257_MOESM6_ESM.pdf]

**Figure 4**

**c**

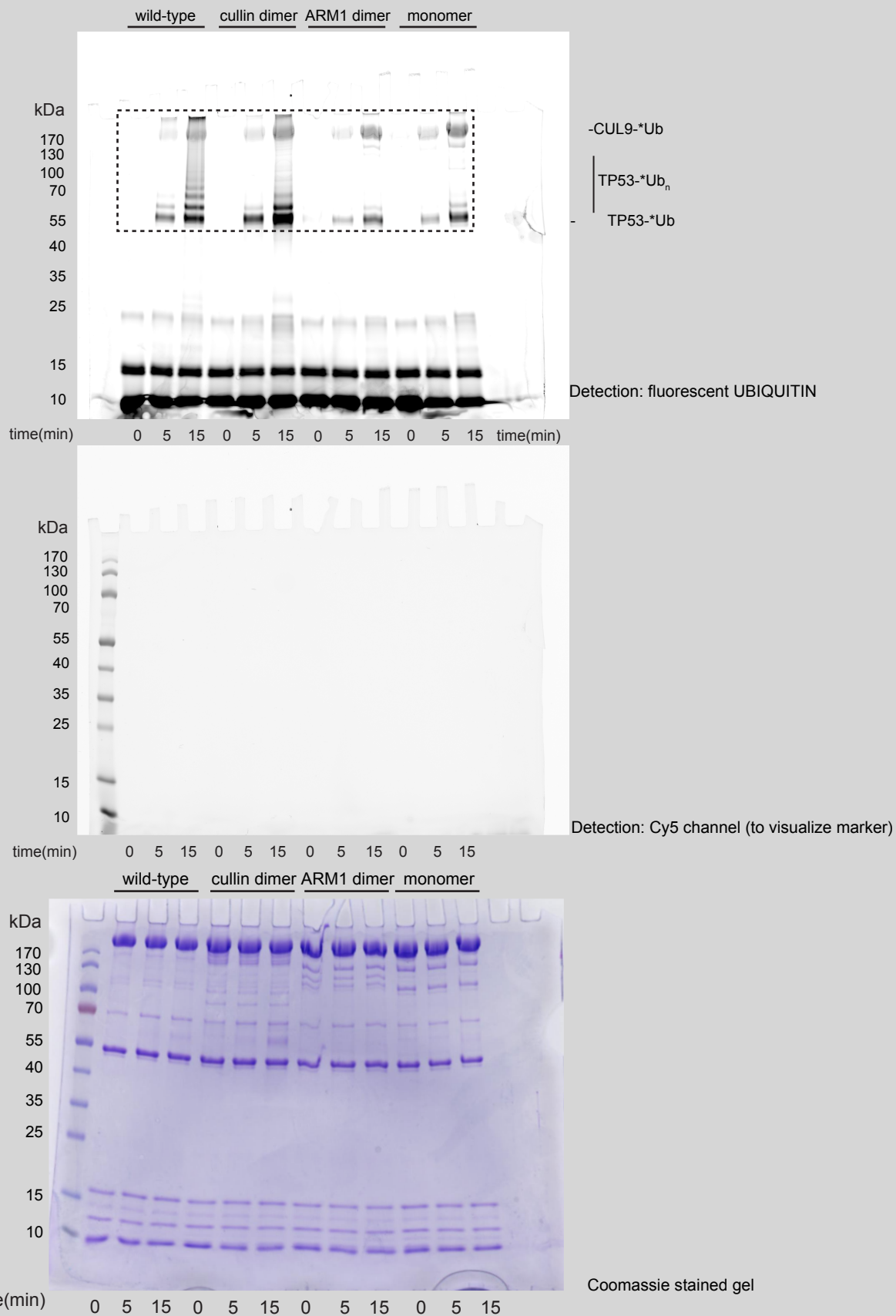

Supplement: Supplementary file 7 — Unprocessed western blots and/or gels. [file 41594_2024_1257_MOESM7_ESM.pdf]

Figure 5

a

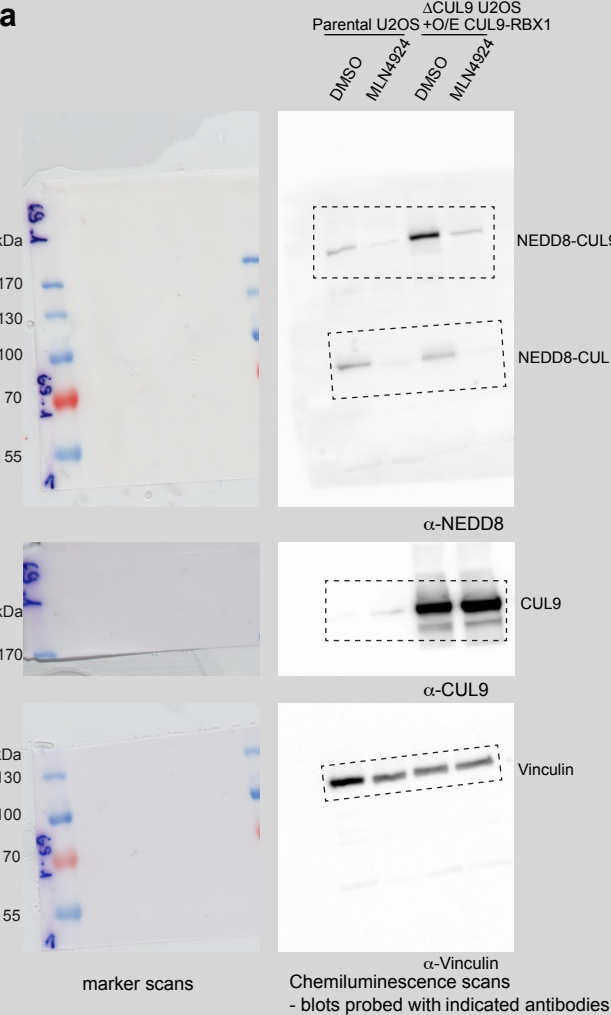

c

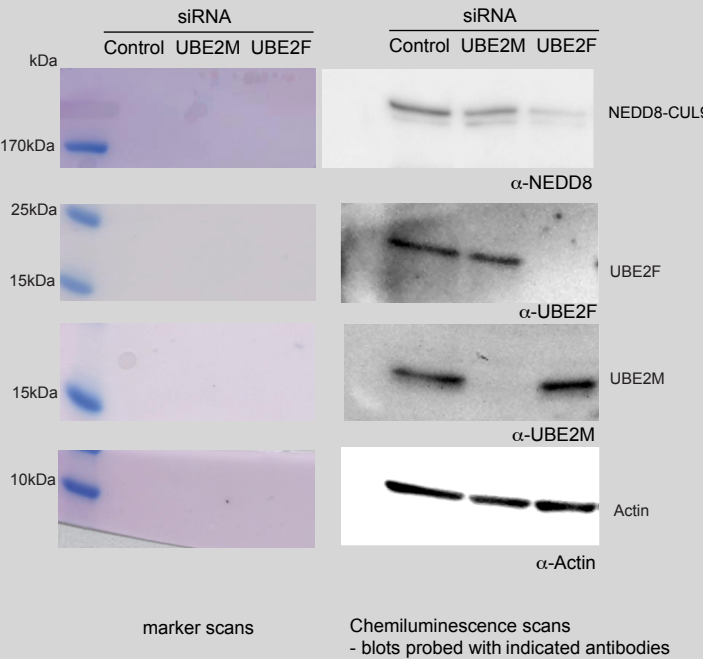

Figure 5  
d-e

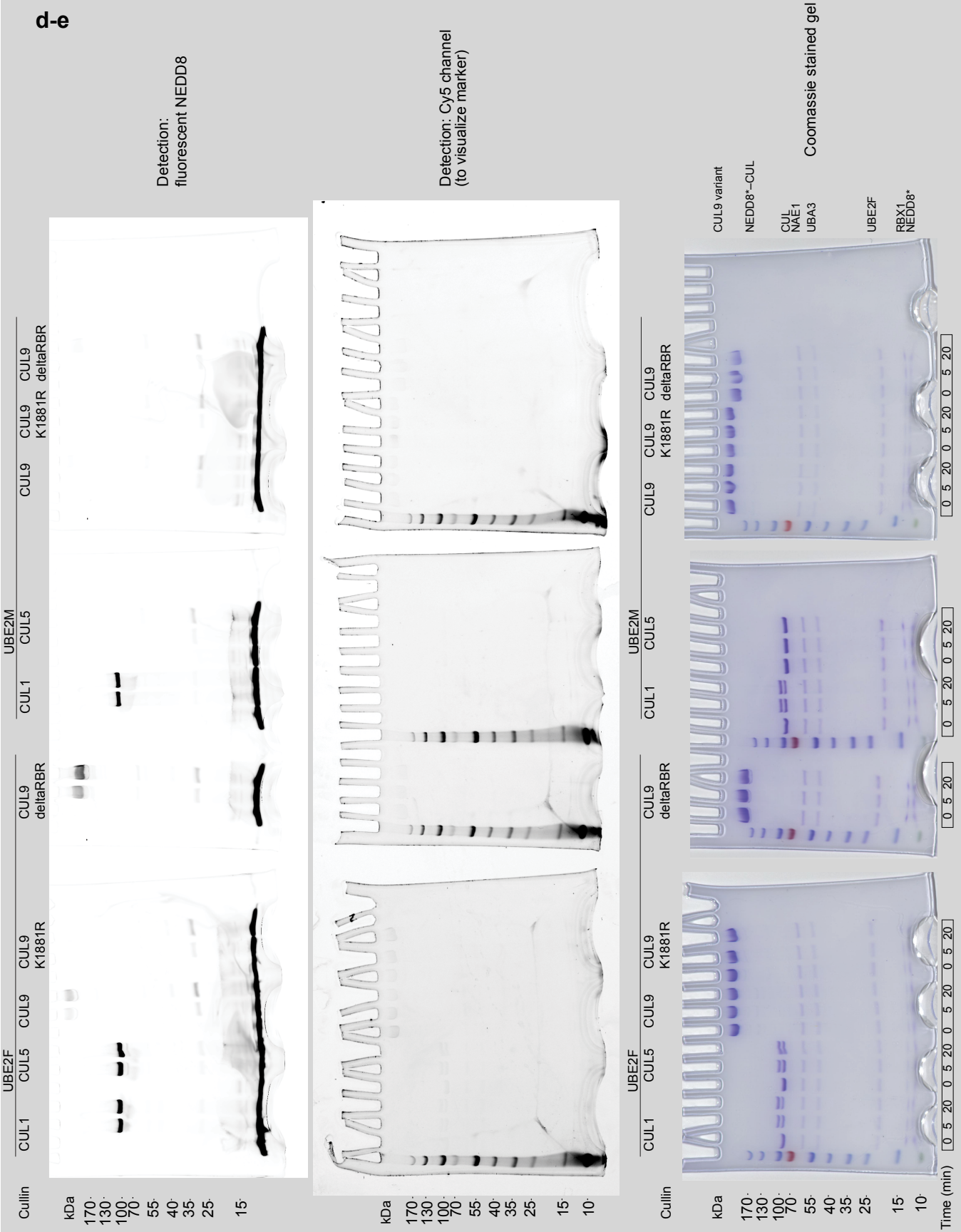

### Figure 5 f-g

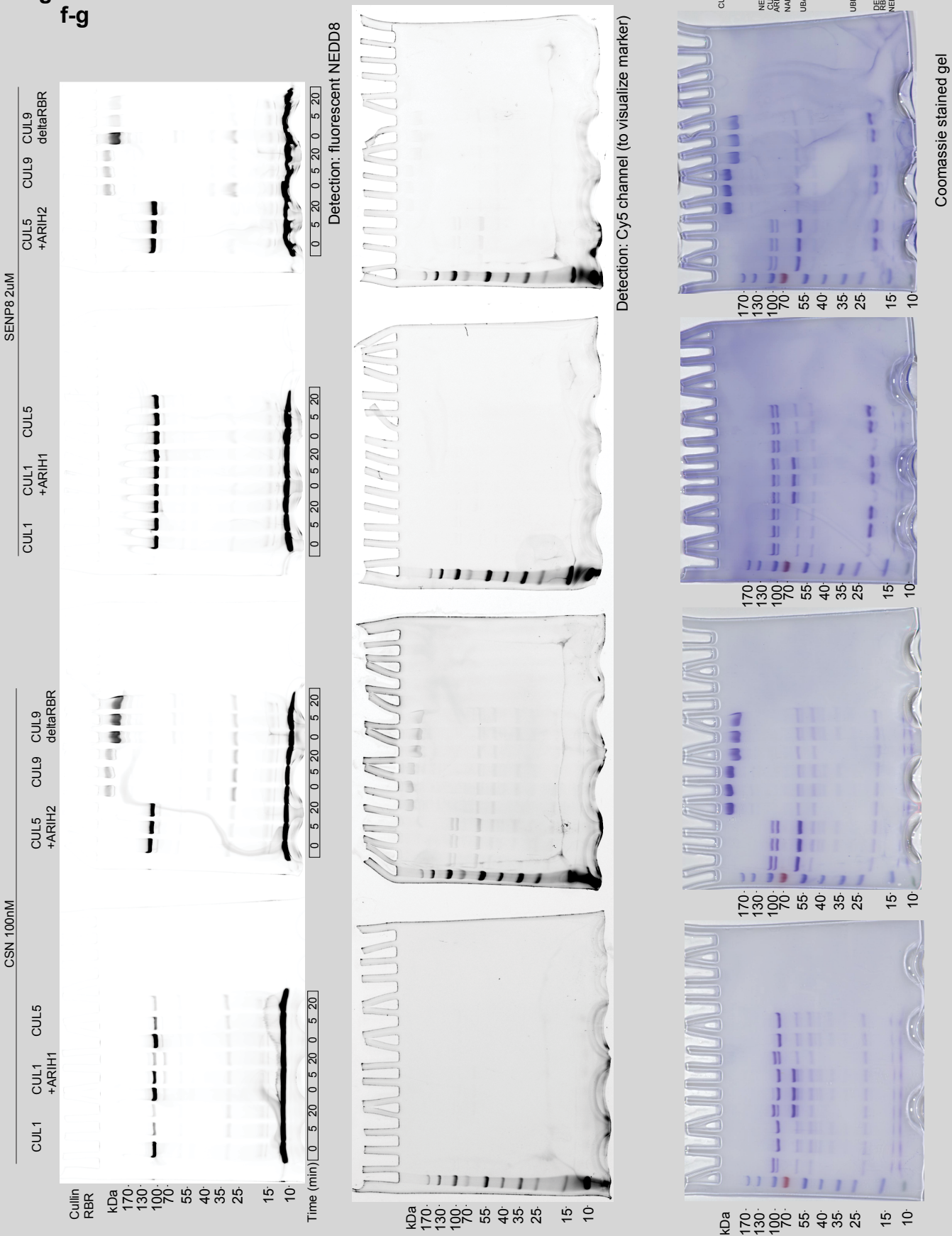

Supplement: Supplementary file 8 — Unprocessed western blots and/or gels. [file 41594_2024_1257_MOESM8_ESM.pdf]

Extended Data Figure 9

a

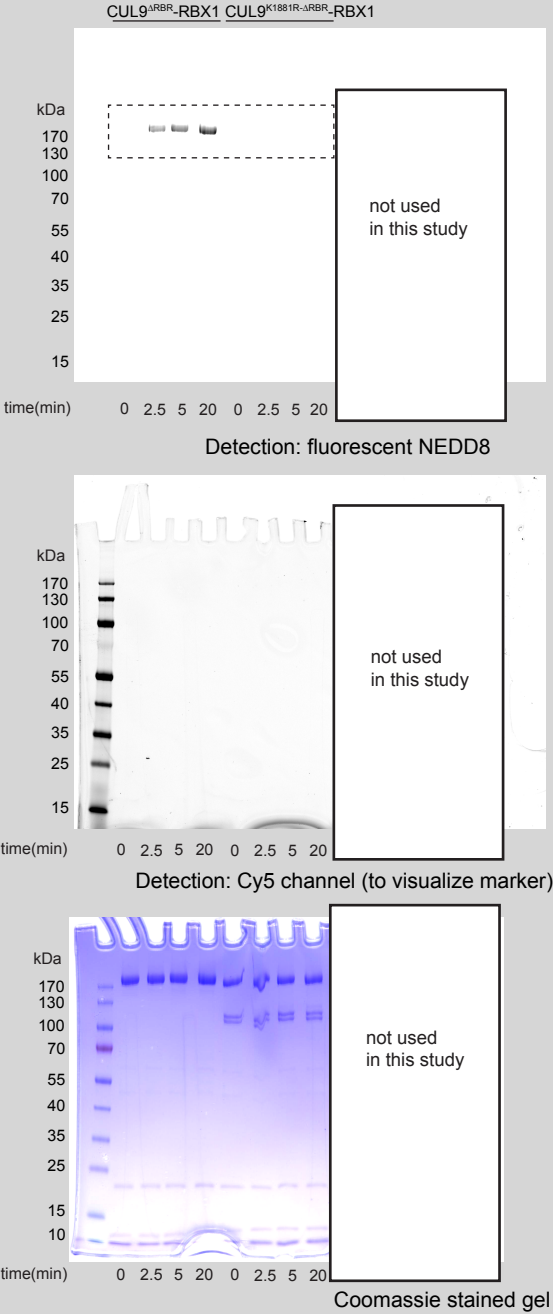

b

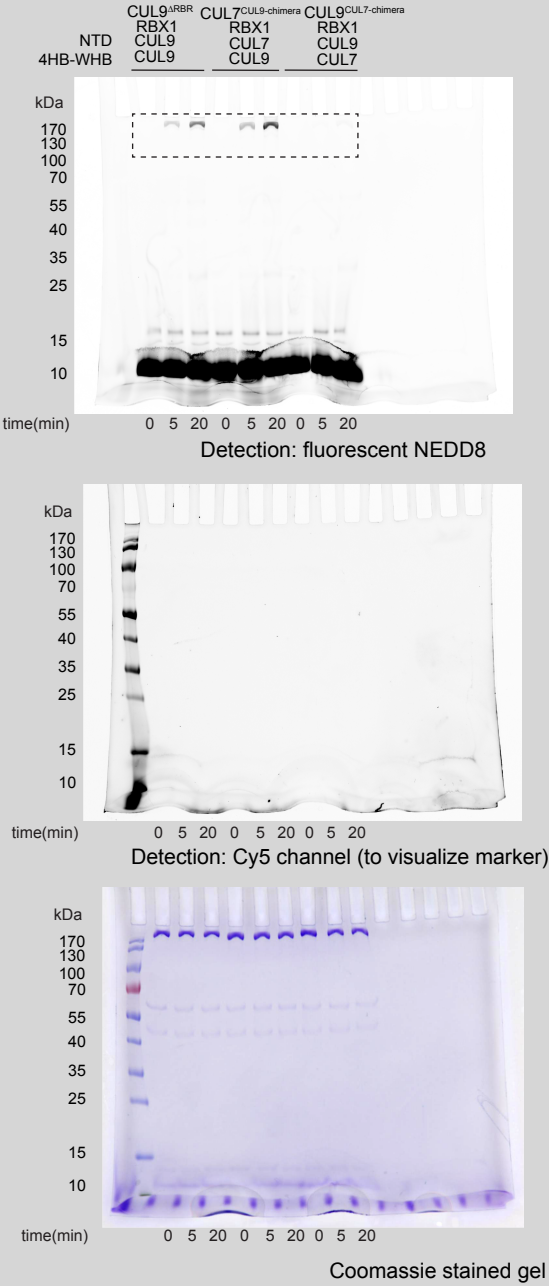

Extended Data Figure 9

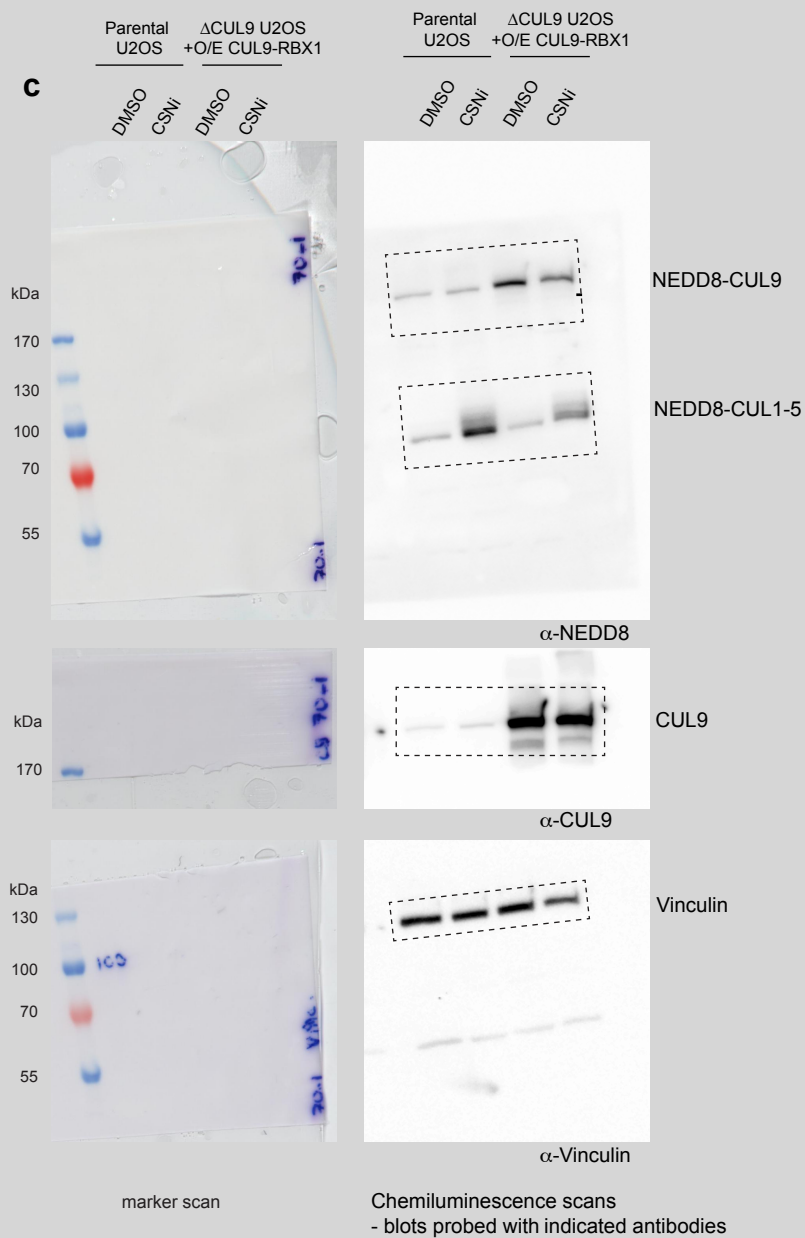

Supplement: Supplementary file 12 — Unprocessed western blots and/or gels. [file 41594_2024_1257_MOESM12_ESM.pdf]
