# Supplementary material for: Noncanonical assembly, neddylation and chimeric cullin–RING/RBR ubiquitylation by the 1.8 MDa CUL9 E3 ligase complex
Source: Nat Struct Mol Biol. 2024 Apr 11;31(7):1083–94. doi: 10.1038/s41594-024-01257-y (PMC11257990; doi:10.1038/s41594-024-01257-y)

Extended Data Figure 6

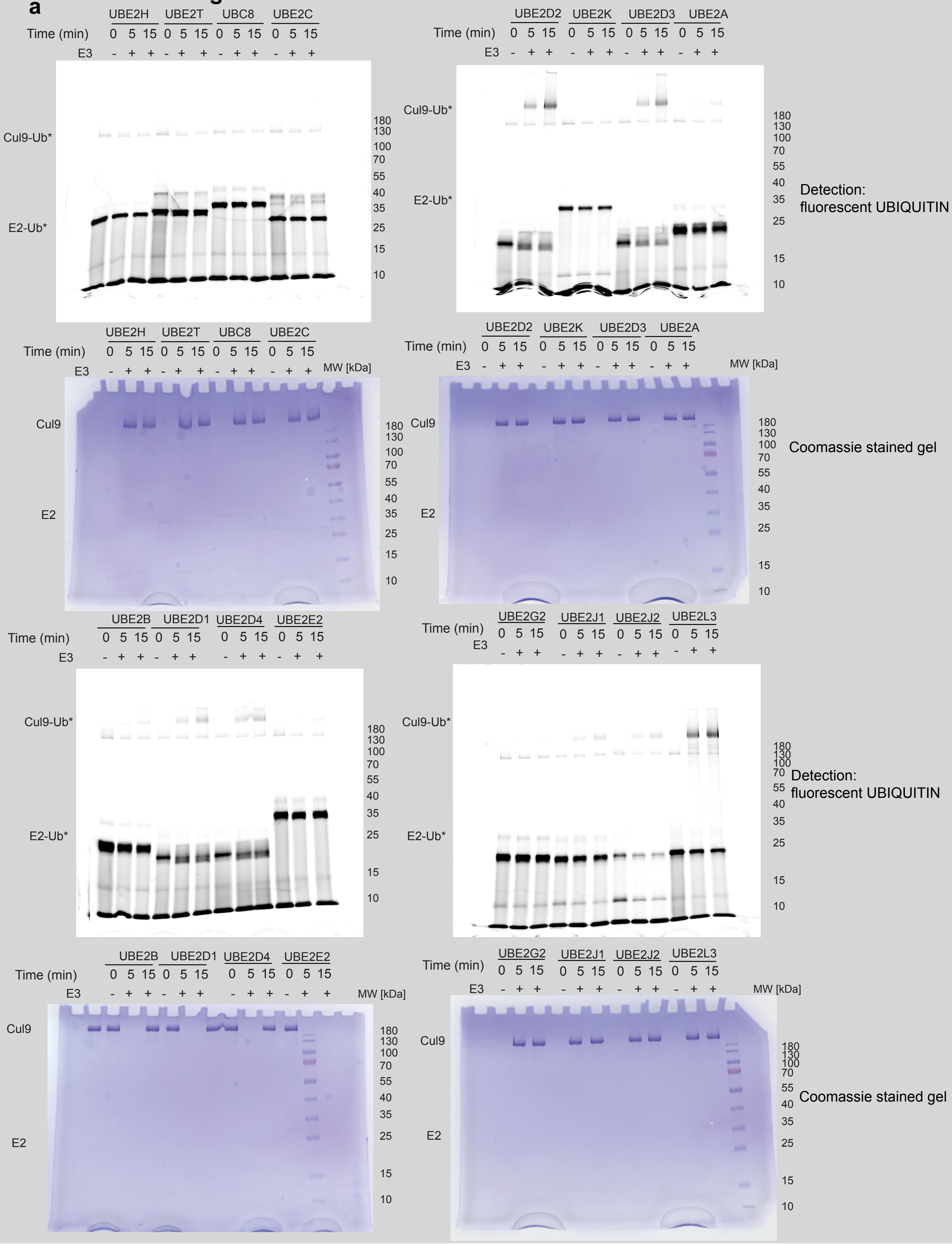

Extended Data Figure 6

a

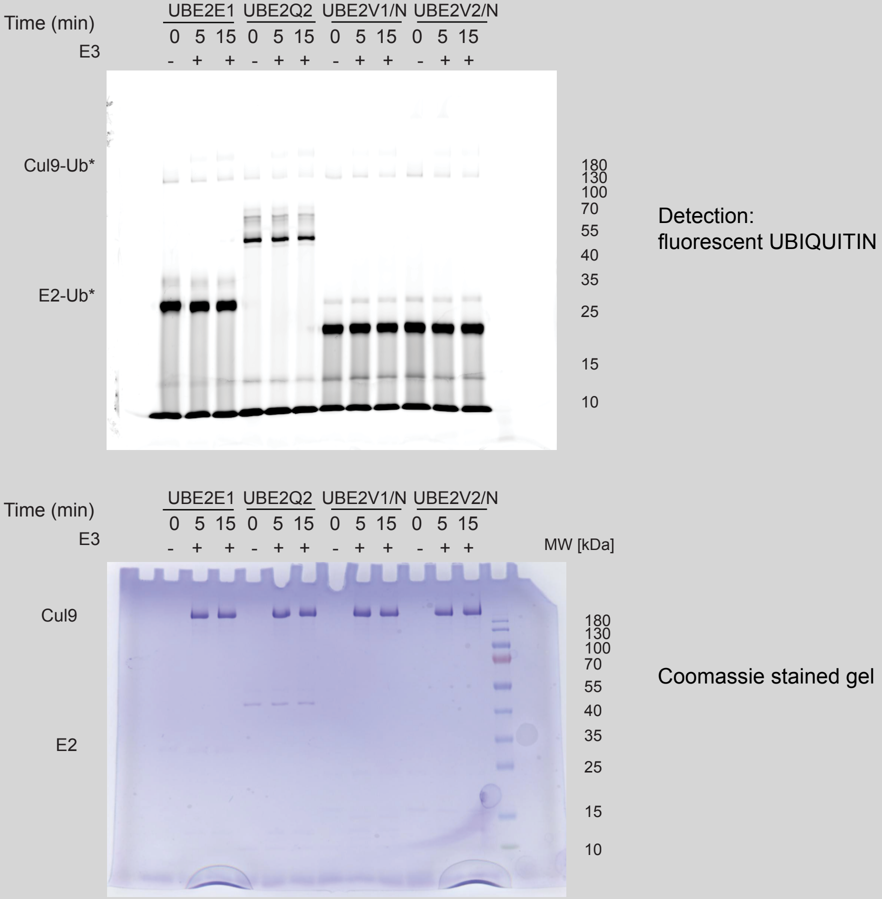

Extended Data Figure 6

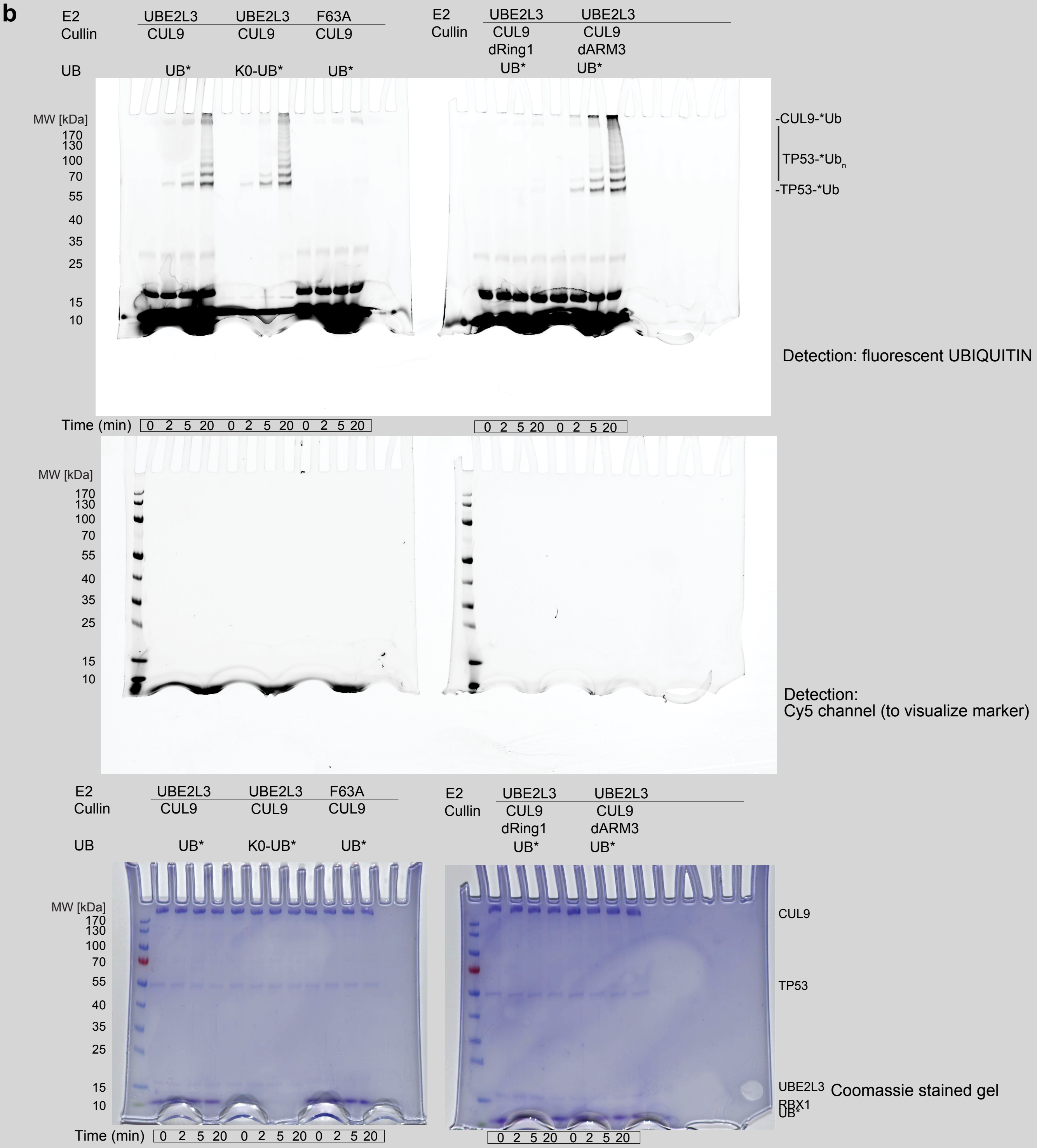

Extended Data Figure 6

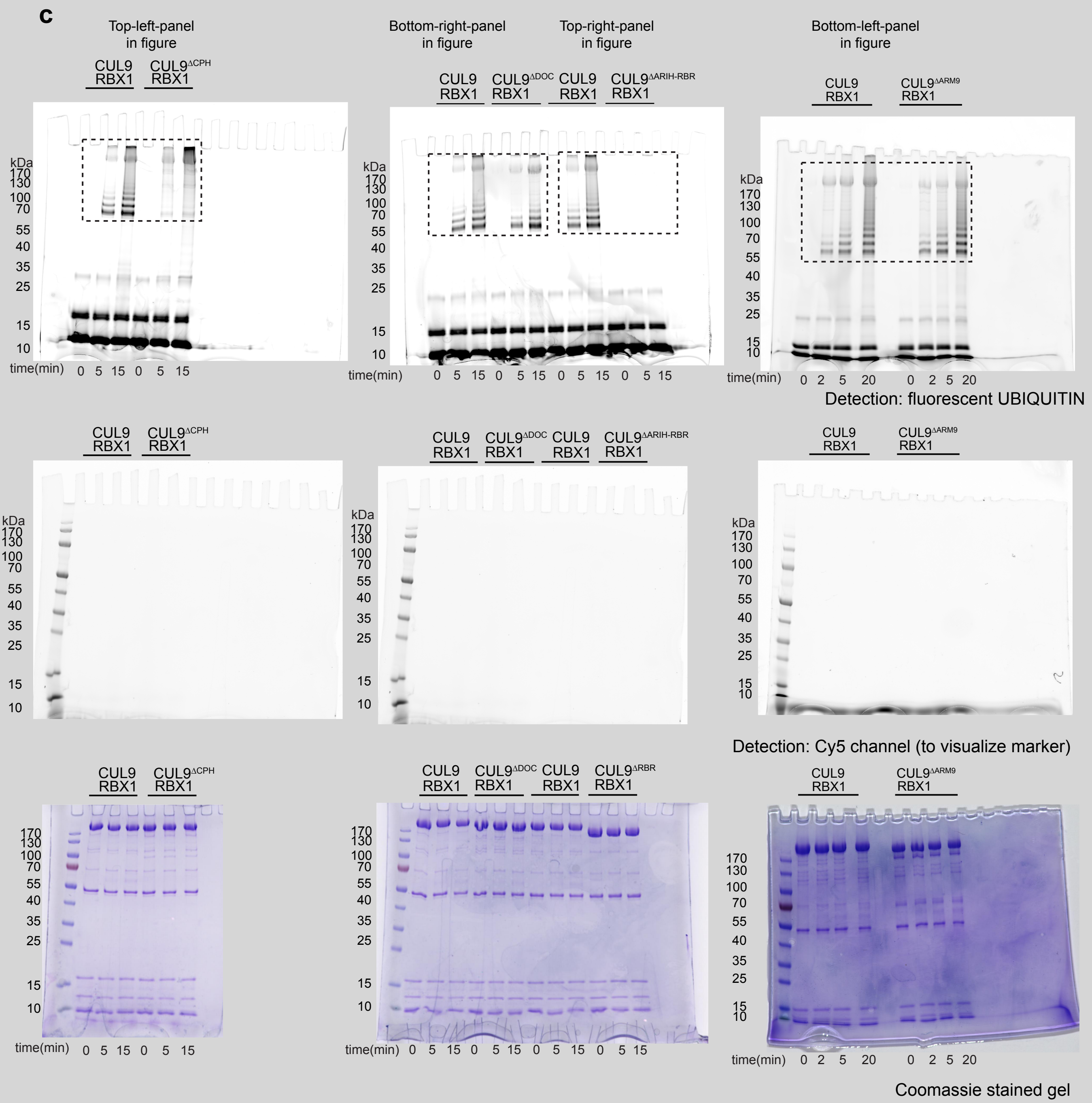

Supplement: Supplementary file 9 — Unprocessed western blots and/or gels. [file 41594_2024_1257_MOESM9_ESM.pdf]
